# Bis(η^2^-ethyl­ene)[aza­nidediylbis(diiso­propyl­phosphine selenide)-κ^2^ *Se*,*Se*′]iridium(III)

**DOI:** 10.1107/S1600536809050156

**Published:** 2009-11-28

**Authors:** Fang-Hui Wu, Lude Lu, Taike Duan, Qian-Feng Zhang

**Affiliations:** a Material Chemistry Laboratory, Nanjing University of Science and Technology, Nanjing 210094, People’s Republic of China; b Institute of Molecular Engineering and Applied Chemsitry, Anhui University of Technology, Ma’anshan, Anhui 243002, People’s Republic of China

## Abstract

In the title compound, [Ir(η^2^-C_2_H_4_)_2_(C_12_H_28_NP_2_Se_2_)], the central Ir atom is chelated by the [N(^i^Pr_2_PSe)_2_]^−^ ligand *via* two Se atoms and is coordinated by two η^2^-ethyl­ene mol­ecules *via* four C atoms in an octa­hedral coordination geometry.

## Related literature

For studies of complexes containing [N(*R*_2_P*Q*)_2_]^−^ (*Q* = S, Se, Te) ligands, see: Ly & Woollins (1998 ▶); Rudler *et al.* (1997 ▶). For metal complexes with [N(*R*_2_P*Q*)_2_]^−^ (*Q* = S, Se, Te) as NMR shift reagents, see: Barkaoui *et al.* (1997 ▶). For related structures, see: Cheung *et al.* (2006 ▶); Kirchmann *et al.* (2008 ▶); Lundquist *et al.* (1990 ▶); Parr *et al.* (1999 ▶). For the C—C bond length in free ethyl­ene, see: Stoicheff (1962 ▶).
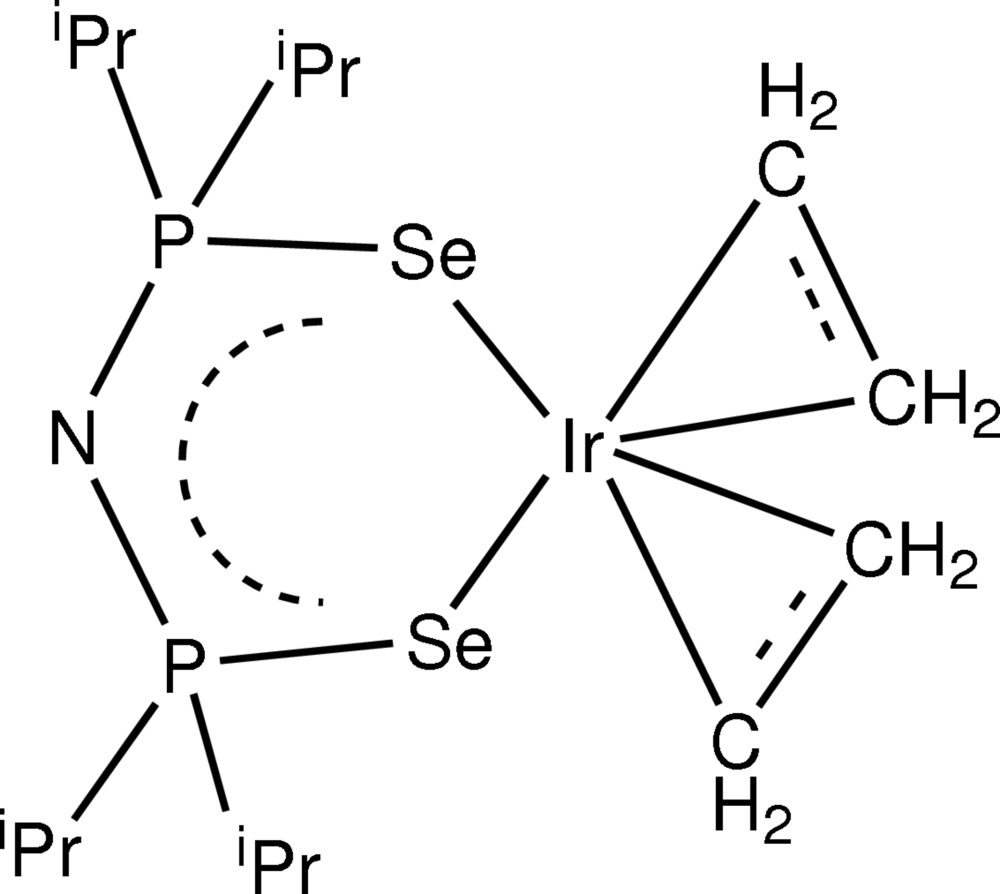


## Experimental

### 

#### Crystal data

[Ir(C_2_H_4_)_2_(C_12_H_28_NP_2_Se_2_)]
*M* *_r_* = 654.52
Triclinic, 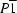

*a* = 9.6965 (1) Å
*b* = 10.3962 (1) Å
*c* = 12.0620 (1) Å
α = 98.511 (1)°
β = 96.055 (1)°
γ = 107.273 (1)°
*V* = 1133.84 (2) Å^3^
*Z* = 2
Mo *K*α radiation
μ = 9.24 mm^−1^
*T* = 296 K
0.30 × 0.21 × 0.16 mm

#### Data collection

Bruker SMART CCD area-detector diffractometer
Absorption correction: multi-scan (*SADABS*; Sheldrick, 1996 ▶) *T*_min_ = 0.168, *T*_max_ = 0.320
15331 measured reflections
5170 independent reflections
4505 reflections with *I* > 2σ(*I*)
*R*_int_ = 0.024

#### Refinement

*R*[*F* ^2^ > 2σ(*F* ^2^)] = 0.028
*wR*(*F* ^2^) = 0.067
*S* = 1.03
5170 reflections
207 parameters
H-atom parameters constrained
Δρ_max_ = 1.39 e Å^−3^
Δρ_min_ = −1.03 e Å^−3^

### 

Data collection: *SMART* (Bruker, 1998 ▶); cell refinement: *SAINT-Plus* (Bruker, 1998 ▶); data reduction: *SAINT-Plus*; program(s) used to solve structure: *SHELXS97* (Sheldrick, 2008 ▶); program(s) used to refine structure: *SHELXL97* (Sheldrick, 2008 ▶); molecular graphics: *SHELXTL* (Sheldrick, 2008 ▶); software used to prepare material for publication: *SHELXTL*.

## Supplementary Material

Crystal structure: contains datablocks I, global. DOI: 10.1107/S1600536809050156/ng2681sup1.cif

Structure factors: contains datablocks I. DOI: 10.1107/S1600536809050156/ng2681Isup2.hkl

Additional supplementary materials:  crystallographic information; 3D view; checkCIF report

## Figures and Tables

**Fig. 1.**
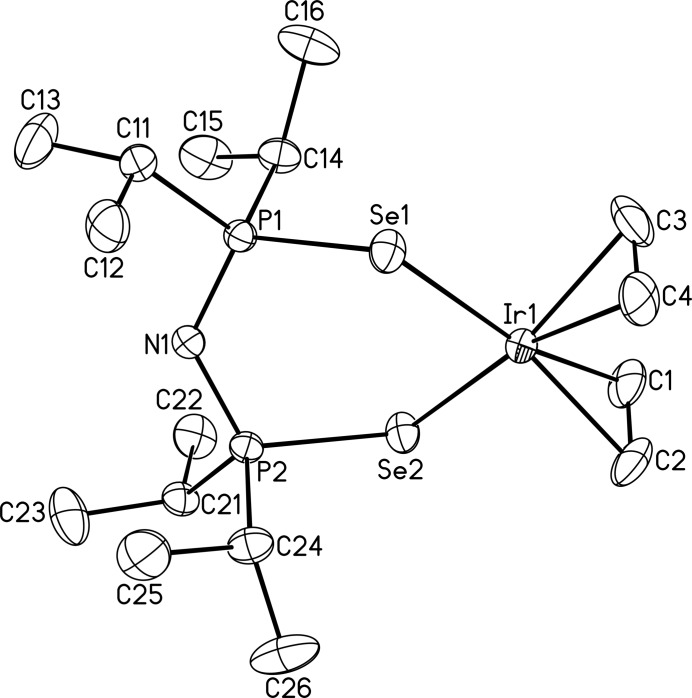
The structure of the title compound, showing the atom-numbering scheme and displacement ellipsoids at the 50% probability level.

## supplementary crystallographic information

### Comment

Imidotetraaryldichalcogenodiphosphinates of general formula [N(*R*_2_PQ)_2_]^-^ (Q = S, Se, Te) have a well defined coordination chemistry, with examples of simple complexes known for elements from each block of the periodic table (Ly & Woollins, 1998). Metal complexes with [N(*R*_2_PQ)_2_]^-^ have been used as catalysts for organic reactions (Rudler *et al.*, 1997) and NMR shift reagents (Barkaoui *et al.*, 1997). Despite the high affinity of [N(*R*_2_PQ)_2_]^-^ for late transition metal ions, their complexes with iridium have not been well explored. The only structurally characterized iridium imidotetraaryldichalcogenodiphosphinates are the half-sandwich complexes [Cp*Ir{N(*R*_2_PQ)_2_}*X*] (*X* = Cl, SCN, SeCN) (Parr *et al.*, 1999). To explore the organometallic chemistry of imidotetraaryldichalcogenodiphosphinates, we are interested to synthesize organoiridium compounds with [N(*R*_2_PQ)_2_]^-^ ligands. We have previously reported the syntheses and crystal structures of iridium imidotetraaryldithiodiphosphinates compounds containing carbonyl, 1,5-cyclooctadiene, and olefin co-ligands (Cheung *et al.*, 2006). We herein describe molecular structure of an organoiridium compound [Ir{N(^i^Pr_2_PSe)_2_}(η^2^-C_2_H_4_)_2_] with imido-tetra-iso-propyldiselenodiphosphinate and ethylene ligands in this paper.

The title compound crystallizes in the triclinic space group P-1. The molecular structure with atomic numbering is depicted in Fig. 1. The central iridium atom is hexacoordinated by one chelating [N(^i^Pr_2_PSe)_2_]^-^ ligand *via* two selenium atoms and two η^2^-ethylene molecules *via* four carbon atoms, thereby forming a distorded octahedral environment. The [N(^i^Pr_2_PSe)_2_]^-^ acts as a chelating ligand through its two selenium atoms to bond the iridium center in a *cis* geometry. Similar to analogue complexes [Ir{N(^i^Pr_2_PS)_2_}(η^2^-C_2_H_4_)_2_] and [Ir{N(Ph_2_PS)_2_}(η^2^-COE)_2_] (COE = cyclooctene) with imido-tetra-iso-propyldiselenodiphosphinate ligands (Cheung *et al.*, 2006), the conformation of the six-membered IrSe_2_P_2_N ring in the title compound is described as a twisted-boat imposed by the non-parallel orientation of the two P—Se bonds. The bite angle of Se(1)—Ir(1)—Se(2) is 99.332 (16)°, the other angles in the chelate ring are Ir(1)—Se(1)—P(1) 109.29 (3)°, Ir(1)—Se(2)—P(2) 110.38 (3)°, Se(1)—P(1)—N(1) 116.89 (14)°, Se(2)—P(2)—N(1) 116.88 (13)°, and P(1)—N(1)—P(2) 127.3 (2)°. The average Ir—Se bond length (av. 2.4601 (5) Å) in the title compound is slightly longer than those in [Cp^*^Ir{N(Ph_2_PSe)_2_}Cl] (av. 2.5139 (5) Å) (Parr *et al.*, 1999) and [Cp^*^Ir{N(Ph_2_PSe)_2_}(SCN)] (av. 2.5159 (8) Å) (Parr *et al.*, 1999). The ethylene molecules bind to the central iridium atom in an η^2^-coordination mode with the small bite angles C(1)—Ir(1)—C(2) and C(1)—Ir(1)—C(2) of 38.1 (2) and 38.4 (2)°, respectively. The average C—C bond length in the title compound (av. 1.398 (9) Å) is longer than that in free ethylene (1.339 Å) (Stoicheff, 1962), but compares with corresponding bond lengths in [Ir{N(^i^Pr_2_PS)_2_}(η^2^-C_2_H_4_)_2_] (av. 1.400 (6) Å) (Cheung *et al.*, 2006), [Ir(PMe_2_Ph)_3_(η^2^-C_2_H_4_)_2_][BF_4_] (av. 1.42 (2) Å) (Lundquist *et al.*, 1990) and [Me_4_N][Ir(*SnB*_11_H_11_)(CO)(η^2^-C_2_H_4_)(PPh_3_)_2_] (av. 1.44 (3) Å) (Kirchmann *et al.*, 2008). The average Ir—C bond length in the title compound (av. 2.133 (5) Å) compares well with those in [Ir{N(^i^Pr_2_PS)_2_}(η^2^-C_2_H_4_)_2_] (av. 2.124 (5) Å) (Cheung *et al.*, 2006), [Ir(PMe_2_Ph)_3_(η^2^-C_2_H_4_)_2_][BF_4_] (av. 2.20 (3) Å) (Lundquist *et al.*, 1990) and [Me_4_N][Ir(*SnB*_11_H_11_)(CO)(η^2^-C_2_H_4_)(PPh_3_)_2_] (av. 2.22 (2) Å) (Kirchmann *et al.*, 2008).

### Experimental

The title compound was prepared according to the literature method (Cheung *et al.*, 2006) and similarly as for [Ir{N(^i^Pr_2_PS)_2_}(η^2^-C_2_H_4_)_2_] using K[N(^i^Pr_2_PSe)_2_] instead of K[N(^i^Pr_2_PS)_2_]. Orange single crystals of the title compound were obtained from the hexane solution at -40 °C.

### Refinement

H atoms were positioned geometrically and refined using a riding model (including free rotation about the ethanol C—C bond), with C—H = 0.95–0.99 Å and with *U*_iso_(H) = 1.2 (1.5 for methyl groups) times *U*_eq_(C).

### Crystal data

**Table:**

[Ir(C_2_H_4_)_2_(C_12_H_28_NP_2_Se_2_)]	*Z* = 2
*M**_r_* = 654.52	*F*(000) = 628
Triclinic, *P*1	*D*_x_ = 1.917 Mg m^−^^3^
Hall symbol: -P 1	Mo *K*α radiation, λ = 0.71073 Å
*a* = 9.6965 (1) Å	Cell parameters from 6523 reflections
*b* = 10.3962 (1) Å	θ = 2.5–26.8°
*c* = 12.0620 (1) Å	µ = 9.24 mm^−^^1^
α = 98.511 (1)°	*T* = 296 K
β = 96.055 (1)°	Prism, orange
γ = 107.273 (1)°	0.30 × 0.21 × 0.16 mm
*V* = 1133.84 (2) Å^3^	

### Data collection

**Table:**

Bruker SMART CCD area-detector diffractometer	5170 independent reflections
Radiation source: fine-focus sealed tube	4505 reflections with *I* > 2σ(*I*)
graphite	*R*_int_ = 0.024
phi and ω scans	θ_max_ = 27.5°, θ_min_ = 2.1°
Absorption correction: multi-scan (*SADABS*; Sheldrick, 1996)	*h* = −12→11
*T*_min_ = 0.168, *T*_max_ = 0.320	*k* = −12→13
15331 measured reflections	*l* = −15→15

### Refinement

**Table:**

Refinement on *F*^2^	Primary atom site location: structure-invariant direct methods
Least-squares matrix: full	Secondary atom site location: difference Fourier map
*R*[*F*^2^ > 2σ(*F*^2^)] = 0.028	Hydrogen site location: inferred from neighbouring sites
*wR*(*F*^2^) = 0.067	H-atom parameters constrained
*S* = 1.03	*w* = 1/[σ^2^(*F*_o_^2^) + (0.0339*P*)^2^ + 0.943*P*] where *P* = (*F*_o_^2^ + 2*F*_c_^2^)/3
5170 reflections	(Δ/σ)_max_ < 0.001
207 parameters	Δρ_max_ = 1.39 e Å^−^^3^
0 restraints	Δρ_min_ = −1.03 e Å^−^^3^

### Special details

**Table:**

Geometry. All e.s.d.'s (except the e.s.d. in the dihedral angle between two l.s. planes) are estimated using the full covariance matrix. The cell e.s.d.'s are taken into account individually in the estimation of e.s.d.'s in distances, angles and torsion angles; correlations between e.s.d.'s in cell parameters are only used when they are defined by crystal symmetry. An approximate (isotropic) treatment of cell e.s.d.'s is used for estimating e.s.d.'s involving l.s. planes.
Refinement. Refinement of *F*^2^ against ALL reflections. The weighted *R*-factor *wR* and goodness of fit *S* are based on *F*^2^, conventional *R*-factors *R* are based on *F*, with *F* set to zero for negative *F*^2^. The threshold expression of *F*^2^ > σ(*F*^2^) is used only for calculating *R*-factors(gt) *etc*. and is not relevant to the choice of reflections for refinement. *R*-factors based on *F*^2^ are statistically about twice as large as those based on *F*, and *R*- factors based on ALL data will be even larger.

### Fractional atomic coordinates and isotropic or equivalent isotropic displacement parameters (Å^2^)

**Table:**

	*x*	*y*	*z*	*U*_iso_*/*U*_eq_	
Ir1	0.183001 (17)	0.291467 (16)	0.217100 (14)	0.03765 (6)	
Se1	0.07448 (5)	0.45184 (4)	0.31713 (4)	0.04549 (12)	
Se2	0.39296 (5)	0.45760 (5)	0.16421 (5)	0.04878 (12)	
P1	0.24433 (11)	0.65130 (11)	0.37985 (9)	0.0347 (2)	
P2	0.35610 (11)	0.65739 (11)	0.16970 (9)	0.0356 (2)	
N1	0.3238 (4)	0.7270 (3)	0.2873 (3)	0.0398 (8)	
C1	0.3029 (7)	0.1529 (6)	0.1735 (6)	0.0703 (16)	
H1A	0.3089	0.2106	0.2419	0.084*	
H1B	0.3632	0.0982	0.1679	0.084*	
C2	0.2036 (7)	0.1485 (6)	0.0795 (5)	0.0737 (18)	
H2A	0.1433	0.2033	0.0852	0.088*	
H2B	0.1976	0.0909	0.0112	0.088*	
C3	0.0789 (7)	0.1508 (6)	0.3164 (6)	0.0790 (19)	
H3A	0.1740	0.2104	0.3255	0.095*	
H3B	0.0517	0.0953	0.3694	0.095*	
C4	−0.0233 (6)	0.1446 (6)	0.2229 (6)	0.0764 (19)	
H4A	0.0039	0.2000	0.1699	0.092*	
H4B	−0.1184	0.0850	0.2138	0.092*	
C11	0.1481 (5)	0.7590 (5)	0.4494 (4)	0.0475 (11)	
H11A	0.1057	0.7149	0.5098	0.057*	
C12	0.0227 (6)	0.7685 (6)	0.3672 (5)	0.0652 (15)	
H12A	−0.0331	0.8161	0.4079	0.098*	
H12B	−0.0393	0.6778	0.3319	0.098*	
H12C	0.0613	0.8175	0.3100	0.098*	
C13	0.2494 (7)	0.9012 (6)	0.5043 (6)	0.086 (2)	
H13A	0.3119	0.9376	0.4521	0.129*	
H13B	0.3080	0.8959	0.5717	0.129*	
H13C	0.1924	0.9601	0.5237	0.129*	
C14	0.3761 (5)	0.6209 (5)	0.4850 (4)	0.0490 (11)	
H14A	0.4022	0.5431	0.4475	0.059*	
C15	0.5195 (6)	0.7394 (7)	0.5211 (6)	0.080 (2)	
H15A	0.5045	0.8113	0.5733	0.121*	
H15B	0.5513	0.7739	0.4554	0.121*	
H15C	0.5927	0.7074	0.5571	0.121*	
C16	0.3095 (7)	0.5757 (8)	0.5874 (5)	0.084 (2)	
H16A	0.3764	0.5451	0.6329	0.126*	
H16B	0.2194	0.5020	0.5619	0.126*	
H16C	0.2909	0.6515	0.6319	0.126*	
C21	0.5261 (5)	0.7694 (5)	0.1388 (4)	0.0465 (11)	
H21A	0.5384	0.7316	0.0624	0.056*	
C22	0.6584 (5)	0.7730 (6)	0.2205 (5)	0.0679 (16)	
H22A	0.7462	0.8198	0.1941	0.102*	
H22B	0.6563	0.6809	0.2245	0.102*	
H22C	0.6560	0.8204	0.2946	0.102*	
C23	0.5204 (7)	0.9147 (6)	0.1376 (7)	0.085 (2)	
H23A	0.4863	0.9469	0.2049	0.128*	
H23B	0.4548	0.9139	0.0719	0.128*	
H23C	0.6164	0.9746	0.1354	0.128*	
C24	0.2058 (5)	0.6298 (5)	0.0541 (4)	0.0510 (12)	
H24A	0.1274	0.5496	0.0641	0.061*	
C25	0.1402 (7)	0.7457 (7)	0.0570 (6)	0.0793 (18)	
H25A	0.2084	0.8238	0.0375	0.119*	
H25B	0.1196	0.7703	0.1319	0.119*	
H25C	0.0513	0.7164	0.0035	0.119*	
C26	0.2453 (7)	0.5916 (9)	−0.0620 (5)	0.092 (2)	
H26A	0.1588	0.5617	−0.1182	0.138*	
H26B	0.2874	0.5190	−0.0616	0.138*	
H26C	0.3148	0.6702	−0.0798	0.138*	

### Atomic displacement parameters (Å^2^)

**Table:**

	*U*^11^	*U*^22^	*U*^33^	*U*^12^	*U*^13^	*U*^23^
Ir1	0.03986 (10)	0.03282 (9)	0.03745 (10)	0.00918 (7)	0.00462 (7)	0.00396 (6)
Se1	0.0369 (2)	0.0379 (2)	0.0589 (3)	0.00759 (18)	0.0148 (2)	0.0044 (2)
Se2	0.0454 (2)	0.0417 (2)	0.0647 (3)	0.01693 (19)	0.0215 (2)	0.0117 (2)
P1	0.0354 (5)	0.0349 (5)	0.0337 (6)	0.0103 (4)	0.0063 (4)	0.0076 (4)
P2	0.0335 (5)	0.0390 (6)	0.0348 (6)	0.0110 (4)	0.0058 (4)	0.0090 (4)
N1	0.046 (2)	0.0352 (19)	0.037 (2)	0.0096 (16)	0.0086 (16)	0.0081 (15)
C1	0.087 (4)	0.050 (3)	0.081 (4)	0.038 (3)	0.023 (4)	−0.007 (3)
C2	0.083 (4)	0.057 (3)	0.069 (4)	0.017 (3)	0.021 (3)	−0.025 (3)
C3	0.090 (4)	0.057 (3)	0.100 (5)	0.015 (3)	0.043 (4)	0.045 (4)
C4	0.067 (4)	0.049 (3)	0.091 (5)	−0.019 (3)	0.030 (3)	0.007 (3)
C11	0.059 (3)	0.043 (2)	0.046 (3)	0.019 (2)	0.020 (2)	0.010 (2)
C12	0.071 (3)	0.060 (3)	0.080 (4)	0.039 (3)	0.021 (3)	0.018 (3)
C13	0.097 (5)	0.054 (3)	0.092 (5)	0.016 (3)	0.021 (4)	−0.019 (3)
C14	0.042 (2)	0.064 (3)	0.042 (3)	0.018 (2)	0.002 (2)	0.015 (2)
C15	0.055 (3)	0.096 (5)	0.066 (4)	−0.004 (3)	−0.014 (3)	0.018 (3)
C16	0.074 (4)	0.129 (6)	0.064 (4)	0.035 (4)	0.015 (3)	0.056 (4)
C21	0.044 (2)	0.051 (3)	0.047 (3)	0.012 (2)	0.018 (2)	0.015 (2)
C22	0.041 (3)	0.068 (4)	0.082 (4)	−0.002 (2)	0.009 (3)	0.015 (3)
C23	0.074 (4)	0.055 (4)	0.135 (6)	0.013 (3)	0.039 (4)	0.042 (4)
C24	0.045 (2)	0.067 (3)	0.040 (3)	0.020 (2)	0.000 (2)	0.006 (2)
C25	0.082 (4)	0.095 (5)	0.072 (4)	0.051 (4)	−0.006 (3)	0.013 (4)
C26	0.077 (4)	0.157 (7)	0.042 (3)	0.056 (5)	−0.006 (3)	−0.005 (4)

### Geometric parameters (Å, °)

**Table:**

Ir1—C3	2.119 (5)	C13—H13A	0.9600
Ir1—C2	2.125 (5)	C13—H13B	0.9600
Ir1—C4	2.141 (5)	C13—H13C	0.9600
Ir1—C1	2.145 (5)	C14—C16	1.524 (7)
Ir1—Se2	2.4590 (5)	C14—C15	1.529 (7)
Ir1—Se1	2.4611 (5)	C14—H14A	0.9800
Se1—P1	2.1975 (11)	C15—H15A	0.9600
Se2—P2	2.2026 (12)	C15—H15B	0.9600
P1—N1	1.595 (3)	C15—H15C	0.9600
P1—C11	1.826 (5)	C16—H16A	0.9600
P1—C14	1.832 (5)	C16—H16B	0.9600
P2—N1	1.599 (4)	C16—H16C	0.9600
P2—C21	1.825 (4)	C21—C22	1.521 (7)
P2—C24	1.832 (5)	C21—C23	1.530 (7)
C1—C2	1.394 (9)	C21—H21A	0.9800
C1—H1A	0.9300	C22—H22A	0.9600
C1—H1B	0.9300	C22—H22B	0.9600
C2—H2A	0.9300	C22—H22C	0.9600
C2—H2B	0.9300	C23—H23A	0.9600
C3—C4	1.401 (9)	C23—H23B	0.9600
C3—H3A	0.9300	C23—H23C	0.9600
C3—H3B	0.9300	C24—C26	1.518 (7)
C4—H4A	0.9300	C24—C25	1.519 (8)
C4—H4B	0.9300	C24—H24A	0.9800
C11—C13	1.517 (7)	C25—H25A	0.9600
C11—C12	1.522 (7)	C25—H25B	0.9600
C11—H11A	0.9800	C25—H25C	0.9600
C12—H12A	0.9600	C26—H26A	0.9600
C12—H12B	0.9600	C26—H26B	0.9600
C12—H12C	0.9600	C26—H26C	0.9600
			
C3—Ir1—C2	98.6 (3)	C11—C12—H12C	109.5
C3—Ir1—C4	38.4 (2)	H12A—C12—H12C	109.5
C2—Ir1—C4	86.8 (2)	H12B—C12—H12C	109.5
C3—Ir1—C1	86.0 (2)	C11—C13—H13A	109.5
C2—Ir1—C1	38.1 (2)	C11—C13—H13B	109.5
C4—Ir1—C1	98.9 (2)	H13A—C13—H13B	109.5
C3—Ir1—Se2	154.8 (2)	C11—C13—H13C	109.5
C2—Ir1—Se2	86.18 (16)	H13A—C13—H13C	109.5
C4—Ir1—Se2	166.4 (2)	H13B—C13—H13C	109.5
C1—Ir1—Se2	82.61 (16)	C16—C14—C15	111.5 (5)
C3—Ir1—Se1	86.27 (17)	C16—C14—P1	112.6 (4)
C2—Ir1—Se1	156.23 (19)	C15—C14—P1	114.1 (4)
C4—Ir1—Se1	82.66 (17)	C16—C14—H14A	105.9
C1—Ir1—Se1	165.18 (18)	C15—C14—H14A	105.9
Se2—Ir1—Se1	99.332 (16)	P1—C14—H14A	105.9
P1—Se1—Ir1	109.29 (3)	C14—C15—H15A	109.5
P2—Se2—Ir1	110.38 (3)	C14—C15—H15B	109.5
N1—P1—C11	108.1 (2)	H15A—C15—H15B	109.5
N1—P1—C14	111.3 (2)	C14—C15—H15C	109.5
C11—P1—C14	110.2 (2)	H15A—C15—H15C	109.5
N1—P1—Se1	116.89 (14)	H15B—C15—H15C	109.5
C11—P1—Se1	104.32 (16)	C14—C16—H16A	109.5
C14—P1—Se1	105.85 (17)	C14—C16—H16B	109.5
N1—P2—C21	108.2 (2)	H16A—C16—H16B	109.5
N1—P2—C24	111.2 (2)	C14—C16—H16C	109.5
C21—P2—C24	110.3 (2)	H16A—C16—H16C	109.5
N1—P2—Se2	116.88 (13)	H16B—C16—H16C	109.5
C21—P2—Se2	104.26 (16)	C22—C21—C23	110.5 (5)
C24—P2—Se2	105.67 (17)	C22—C21—P2	112.0 (3)
P1—N1—P2	127.3 (2)	C23—C21—P2	111.8 (3)
C2—C1—Ir1	70.2 (3)	C22—C21—H21A	107.4
C2—C1—H1A	120.0	C23—C21—H21A	107.4
Ir1—C1—H1A	49.8	P2—C21—H21A	107.4
C2—C1—H1B	120.0	C21—C22—H22A	109.5
Ir1—C1—H1B	169.8	C21—C22—H22B	109.5
H1A—C1—H1B	120.0	H22A—C22—H22B	109.5
C1—C2—Ir1	71.7 (3)	C21—C22—H22C	109.5
C1—C2—H2A	120.0	H22A—C22—H22C	109.5
Ir1—C2—H2A	48.3	H22B—C22—H22C	109.5
C1—C2—H2B	120.0	C21—C23—H23A	109.5
Ir1—C2—H2B	168.3	C21—C23—H23B	109.5
H2A—C2—H2B	120.0	H23A—C23—H23B	109.5
C4—C3—Ir1	71.7 (3)	C21—C23—H23C	109.5
C4—C3—H3A	120.0	H23A—C23—H23C	109.5
Ir1—C3—H3A	48.3	H23B—C23—H23C	109.5
C4—C3—H3B	120.0	C26—C24—C25	111.0 (5)
Ir1—C3—H3B	168.3	C26—C24—P2	112.6 (4)
H3A—C3—H3B	120.0	C25—C24—P2	114.7 (4)
C3—C4—Ir1	69.9 (3)	C26—C24—H24A	105.9
C3—C4—H4A	120.0	C25—C24—H24A	105.9
Ir1—C4—H4A	50.1	P2—C24—H24A	105.9
C3—C4—H4B	120.0	C24—C25—H25A	109.5
Ir1—C4—H4B	170.1	C24—C25—H25B	109.5
H4A—C4—H4B	120.0	H25A—C25—H25B	109.5
C13—C11—C12	110.4 (5)	C24—C25—H25C	109.5
C13—C11—P1	112.6 (4)	H25A—C25—H25C	109.5
C12—C11—P1	111.3 (3)	H25B—C25—H25C	109.5
C13—C11—H11A	107.5	C24—C26—H26A	109.5
C12—C11—H11A	107.5	C24—C26—H26B	109.5
P1—C11—H11A	107.5	H26A—C26—H26B	109.5
C11—C12—H12A	109.5	C24—C26—H26C	109.5
C11—C12—H12B	109.5	H26A—C26—H26C	109.5
H12A—C12—H12B	109.5	H26B—C26—H26C	109.5
			
C3—Ir1—Se1—P1	131.1 (2)	C1—Ir1—C3—C4	−109.8 (4)
C2—Ir1—Se1—P1	−126.0 (4)	Se2—Ir1—C3—C4	−173.1 (3)
C4—Ir1—Se1—P1	169.5 (2)	Se1—Ir1—C3—C4	82.9 (4)
C1—Ir1—Se1—P1	72.3 (6)	C2—Ir1—C4—C3	108.1 (4)
Se2—Ir1—Se1—P1	−24.11 (4)	C1—Ir1—C4—C3	71.8 (4)
C3—Ir1—Se2—P2	−121.0 (4)	Se2—Ir1—C4—C3	167.4 (6)
C2—Ir1—Se2—P2	136.9 (2)	Se1—Ir1—C4—C3	−93.3 (4)
C4—Ir1—Se2—P2	77.5 (7)	N1—P1—C11—C13	−57.8 (5)
C1—Ir1—Se2—P2	175.02 (19)	C14—P1—C11—C13	63.9 (5)
Se1—Ir1—Se2—P2	−19.84 (4)	Se1—P1—C11—C13	177.1 (4)
Ir1—Se1—P1—N1	59.94 (15)	N1—P1—C11—C12	66.7 (4)
Ir1—Se1—P1—C11	179.16 (16)	C14—P1—C11—C12	−171.6 (3)
Ir1—Se1—P1—C14	−64.58 (17)	Se1—P1—C11—C12	−58.3 (4)
Ir1—Se2—P2—N1	56.66 (15)	N1—P1—C14—C16	168.3 (4)
Ir1—Se2—P2—C21	175.99 (16)	C11—P1—C14—C16	48.5 (5)
Ir1—Se2—P2—C24	−67.67 (17)	Se1—P1—C14—C16	−63.7 (5)
C11—P1—N1—P2	−148.8 (3)	N1—P1—C14—C15	39.8 (5)
C14—P1—N1—P2	90.1 (3)	C11—P1—C14—C15	−80.0 (5)
Se1—P1—N1—P2	−31.6 (3)	Se1—P1—C14—C15	167.8 (4)
C21—P2—N1—P1	−147.9 (3)	N1—P2—C21—C22	69.1 (4)
C24—P2—N1—P1	90.8 (3)	C24—P2—C21—C22	−169.0 (4)
Se2—P2—N1—P1	−30.7 (3)	Se2—P2—C21—C22	−55.9 (4)
C3—Ir1—C1—C2	109.3 (4)	N1—P2—C21—C23	−55.5 (5)
C4—Ir1—C1—C2	73.1 (4)	C24—P2—C21—C23	66.4 (5)
Se2—Ir1—C1—C2	−93.2 (3)	Se2—P2—C21—C23	179.4 (4)
Se1—Ir1—C1—C2	168.2 (5)	N1—P2—C24—C26	168.1 (5)
C3—Ir1—C2—C1	−72.2 (4)	C21—P2—C24—C26	48.1 (5)
C4—Ir1—C2—C1	−108.8 (4)	Se2—P2—C24—C26	−64.1 (5)
Se2—Ir1—C2—C1	82.9 (3)	N1—P2—C24—C25	39.9 (5)
Se1—Ir1—C2—C1	−172.5 (3)	C21—P2—C24—C25	−80.2 (5)
C2—Ir1—C3—C4	−73.7 (4)	Se2—P2—C24—C25	167.7 (4)
